# Do Glasses Modulate Age Perception?

**DOI:** 10.1177/2041669520953457

**Published:** 2020-08-26

**Authors:** Nicolas M. Brunet, Jonathan Sharp

**Affiliations:** Department of Psychology and Neuroscience, Millsaps College, Jackson, Mississippi, United States

**Keywords:** faces, glasses, sunglasses, age perception, eyewear, eyeglasses

## Abstract

No formal studies have reported how glasses influence age perception, except for a London Vision Clinic survey that found that people over 45 look 5 or more years older when wearing eyeglasses. To investigate the effect of eyeglasses and sunglasses on age perception while controlling for age and interpersonal differences, we digitally manipulated the photographs of faces of 50 young adults, to create two age conditions (young and old) and three eyewear conditions (no glasses, eyeglasses, and sunglasses). Participants then estimated the age of the faces, displayed in random order. Contrary to the generally accepted beliefs that wearing eyeglasses makes you look older and wearing sunglasses make you look younger, our results suggest that the effect of glasses on age perception is rather small.

For a society obsessed with beauty and youthful looks, it is surprising that no formal study has ever been conducted to investigate how eyewear affects age perception. After all, according to the Vision Council of America, about half of all women and 42% of men wear eyeglasses. How glasses alter perception has been investigated along many dimensions such as physical attractiveness and intelligence (Lundberg & Sheehan, 1994), and identity (Kramer & Ritchie, 2016), among others. Surprisingly, age was not one of them. The only study, not published in a peer-reviewed journal, was reported by the *Daily Mail* on September 21, 2010, and its content was quickly shared by other news sources. The study was based upon a survey, commissioned by the London Vision Clinic, where participants were either given 10 pictures of people with eyeglasses or 10 pictures of the same people without glasses, and asked to guess their ages. The study reported that people wearing eyeglasses were perceived as 3.3 years older; glasses-wearers over 45 were thought to be 5 years older.

Although those numbers seem reasonable, the topic merits a more formal approach. To that extent, we randomly selected 25 male and 25 female faces with neutral emotional expression (see examples at bottom Figure 1A) from the publicly available Karolinska Directed Emotional faces database (Lundqvist et al., 1998). We then used FaceApp (Wireless Lab, Skolkovo, Russia) to apply an aging filter to make the faces look older (examples at bottom Figure 1C). The same application was used to add generic eyeglasses (examples at top of Figure 1A and C), while a different application, *Stylish Sun Glasses*, was used to add sunglasses (examples at top of Figure 1B and D). Together, the original and doctored photographs constituted a stimuli set of 300 images. Twenty-one college-aged men and women rated the images, displayed in random order, presented at the center (17° × 23° of visual angle) of a Dell 19 in. monitor. More specifically, the participants reported the estimated age of each face using the keyboard. The time limit for each image was 5 seconds. To analyze the data, we simply averaged the responses for each participant across each of six conditions: Two Age Groups (old and young) × Three Eyewear Conditions (eyeglasses, no-glasses, and sunglasses). We then used a paired *t* test, to probe significance levels between the no-glasses and either sunglasses or eyeglasses condition for each of the two age conditions. The results indicate that faces of young adults were perceived as slightly (1.3 years) but significantly, older when eyeglasses were added (Figure 1A). Adding sunglasses, on the other hand, did not alter the perceived age (Figure 1B).

**Figure 1.**
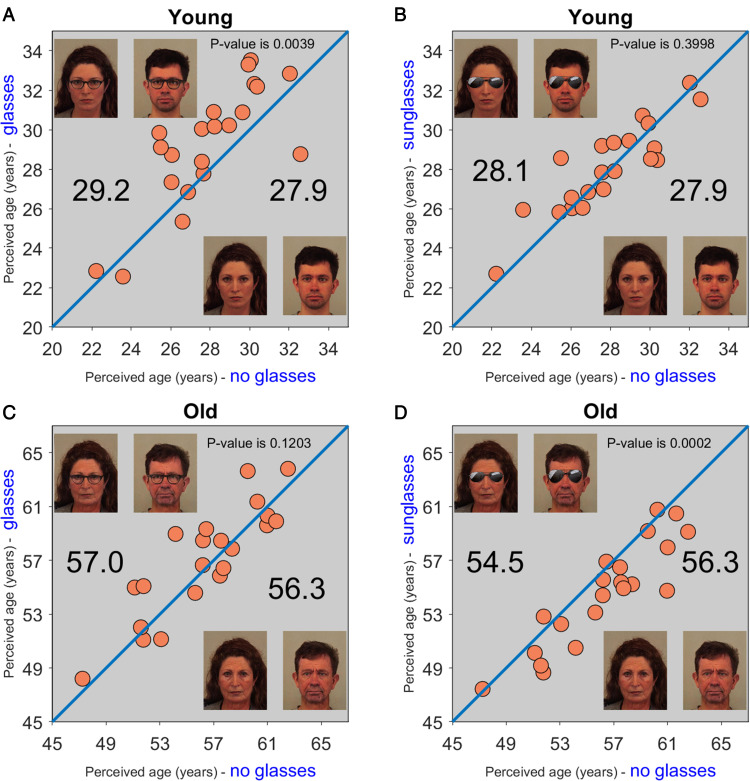
Modulation of Age Perception by Eyewear. A: Each dark orange filled circle represents the result from one participant, with *x* and *y*-coordinates, respectively, representing the values obtained for two conditions: *no glasses* (examples stimuli in bottom right corner) and *eyeglasses* (examples stimuli in top left corner). Data points above the identity line represent data from participants who perceived faces with eyeglasses as older compared with those without glasses. The statistical significance between the two conditions is expressed as a *p* value, shown at the top of the panel. The average perceived age for each condition is displayed at the left (*eyeglasses*) and right (*no glasses*) of the equality line. B: The same as for Panel A, but comparing the *no glasses* (*x*-axis) and *sunglasses* (*y*-axis) conditions. C and D: Similar to, respectively, A and B, but for faces that were manipulated to look older using an aging filter.

The results obtained with faces digitally altered to look older were remarkably different: adding glasses, in this case, did not alter the perceived age (Figure 1C), whereas adding sunglasses significantly reduced the perceived age with 1.8 years (Figure 1D). Statistics (two-way repeated measures analysis of variance) also show that there was a significant interaction between the independent variables (age of face and spectacle type), affecting the perceived age (*p* = .014).

The notion that sunglasses make older people look younger is not that surprising as the darkness of the glasses helps to hide the wrinkles around the eyes that show earlier signs of aging. We do not have a straightforward explanation for why our results contradict with those conducted, albeit not peer-reviewed, by the London Vision Eye Clinic, which received a lot of news coverage. A possible explanation is that participants either rated 10 images of faces with eyeglasses or 10 without glasses, which unlike our randomized and controlled experiment, might cause priming effects. It is also noteworthy that the London Vision Eye clinic provides laser eye surgery, which implies a conflict of interest. When posing with eyeglasses, the conditions (facial expression, lighting, makeup, etc.) might have been different, to look less flattering, compared with posing without glasses. Our study was controlled to avoid such a bias (unchanged facial expression and environmental conditions and addition of generic glasses); at the same time, we acknowledge that there is a possibility that the digitally modified stimuli do not necessarily generalize to real faces. In addition, publicly available face databases, such as the one we used, mainly contain Caucasian faces, where a more diverse face database is desired. Other potential shortcomings are the use of only one style of eyeglasses and sunglasses, and reliance of a participant pool consisting of young adults, which can potentially introduce an own-age bias. A follow-up study might reveal whether this is the case, and whether the reported findings can be extrapolated to older participants. In conclusion, our study suggest that eyewear hardly modulates how we perceive age, which is important because wearing eyeglasses can significantly impact one’s self-esteem (Harris et al., 1982). Our results are also a reminder of how non-peer reviewed research, funded by entities with specific interests, might shape public perception.
